# Development of an Intervention Aimed at Increasing Awareness and Acknowledgement of Victimisation and Its Consequences Among People with Severe Mental Illness

**DOI:** 10.1007/s10597-021-00776-y

**Published:** 2021-01-29

**Authors:** Wendy M. M. Albers, Yolanda A. M. Nijssen, Diana P. K. Roeg, Inge M. B. Bongers, Jaap van Weeghel

**Affiliations:** 1 Tranzo Scientific Center for Care and Wellbeing, Tilburg School of Social and Behavioral Sciences, Tilburg University, PO Box 90153, 5000 LE Tilburg, The Netherlands; 2 Kwintes Housing and Rehabilitation Services, Laan van Vollenhove 3213, 3706 AR Zeist, The Netherlands; 3 Parnassia Psychiatric Institute, Monsterseweg 93, 2553 RJ Den Haag, The Netherlands; 4 GGzE Mental Health Care Institute Eindhoven, Dr. Poletlaan 40, 5626 ND Eindhoven, The Netherlands; 5 Phrenos Center of Expertise, Da Costakade 45, 3521 VS Utrecht, The Netherlands

**Keywords:** Staff training, Rehabilitation, Victimisation, Social participation, Severe mental illness, Discrimination

## Abstract

Individuals with severe mental illness have a significant risk of (anticipated) discrimination and (criminal) victimisation, which is not structurally and systematically addressed by mental health practitioners. The aim of this study was to develop and pilot an intervention which supports professionals to address victimisation and its consequences, in order to reinforce safe social participation and improve recovery. Following the rehabilitation and positive risk management literature, in addition to current practice, intervention components were developed in two focus groups and four subsequent expert meetings. The intervention was piloted in two outpatient teams before being finalised. The Victoria intervention includes positive risk management, focusing on clients’ narratives and strengths, and awareness of unsafe (home) environments: it comprises four steps: exploring issues with social participation, analysing victimisation experiences, clarifying the context of these experiences, and determining future steps, including victimisation-sensitive rehabilitation planning and optional trauma treatment. Future research should further test this intervention.

## Introduction

The shift in mental healthcare from hospital-based toward community-based treatment has placed a stronger focus on the rehabilitation and recovery of individuals with severe mental illness (SMI) (Anthony et al. 2002; Drake et al. 2003). Despite several positive developments that brought forth a greater focus on an inclusive society for people with SMI, including goal attainment (Swildens et al. 2011) and employment (Michon et al. 2014), social participation remains much lower in people with SMI than in the general population. Only 10–20% of such individuals hold down a paid job and around 75–85% have neither paid nor voluntary work (Bond 2004; Marwaha and Johnson 2004). Furthermore, the social networks of people with SMI tend to be smaller and less satisfactory than those of the population as a whole (Gayer-Anderson and Morgan 2013; Koenders et al. 2017; Macdonald et al. 2000; Visentini et al. 2018).

Along with the rise of community mental health care came a growing emphasis on the risks that individuals with SMI have to deal with in participating in that community, such as discrimination and victimisation (Kelly and McKenna 2004). Discrimination is the behavioural aspect of the public stigma attached to mental illness and is defined as being treated in a negative way because of this mental illness (Corrigan 2000). Victimisation is the process of being victimised, and this may be of a violent crime (sexual assault, physical assault) or a non-violent crime such as a property crime (theft, burglary), a digital crime (identity fraud or hacking), or other types of (emotional) abuse or social exploitation (Latalova et al. 2014; Perese 2007). In this study, it always involves recent victimisation. Many clients experience discrimination because of their psychiatric diagnosis, even on a daily basis (Brohan et al. 2010; Lasalvia et al. 2013). Criminal victimisation is also highly prevalent in individuals with SMI; they are considerably more likely to be a victim of crime than others in the community (Brekke et al. 2001; Kamperman et al. 2014; Kelly and McKenna 1997; Latalova et al. 2014; Teplin et al. 2005; Walsh et al. 2003). A large Dutch study identified that Dutch outpatients had six times more incidents than the rest of the population in the previous year (Kamperman et al. 2014). For personal crimes (e.g., sexual harassment/assault, violence, physical assault), the rate is almost 14 times higher than the rate for the rest of the population (Kamperman et al. 2014). Moreover, the majority of perpetrators are familiar to the victim (e.g., a family member, roommate, or neighbour) (Meijwaard et al. 2015). Victimisation, including (anticipated) discrimination, may lead to a vicious cycle of stressful events that are associated with an increase in psychiatric symptoms, substance abuse, an elevated chance of offending, social isolation, a loss of confidence, and even a lower quality of life (Lasalvia et al. 2013; Link and Phelan 2006; Perese 2007; Perlick 2001; Silver et al. 2011).

Several studies have also negatively linked victimisation and discrimination to recovery (Perese 2007) and, more specifically, to social participation (Fitzgerald et al. 2005; Silver 2002). The ‘why-try’ effect, a process of demoralisation among service users, was defined as the effect of perceived stigma and self-stigma and subsequent decreased self-esteem and self-efficacy (Corrigan et al. 2009). This leads them to become discouraged or demoralised about pursuing actions that could advance their recovery process (Corrigan et al. 2009). In addition, clients who do not engage in meaningful daily activities tend to experience more victimisation than clients who do (Fitzgerald et al. 2005). The relationship between victimisation, including (anticipated) discrimination, and social participation tends to be a reciprocal one. Victims of violence tend to acquire problems with maintaining meaningful relationships (Kluft et al. 2000). Moreover, experiences of being rejected can instigate anticipated stigmatisation, and can thus discourage clients from pursuing their rehabilitation goals and wishes (Corrigan et al. 2009), or even lead them to refraining and retracting from participation in community life (Thornicroft 2006).

In outpatient mental health care, professionals have the statutory duty to work with other organisations, partners, and clients’ social environment to identify and address victimisation (WGBO 2019). However, many victimisation incidents remain un-identified and the impact on participation is hardly addressed (Perese 2007). One of the reasons for this is that professionals have almost no tools to systematically address the impact of recent victimisation related to participation, besides interventions for childhood and/or severe trauma (Holley et al. 2016; Walsh et al. 2003). Consequently, interventions on recent victimisation experiences are only minimally integrated into treatment and rehabilitation plans (Dewa et al. 2003; van Weeghel et al. 2011). Paradoxically, addressing recent victimisation is often even seen as increasing the risk of a relapse and therefore preferably evaded (Holley et al. 2016). However, research has shown that the opposite is true; discussing the impact of victimisation experiences can benefit a person’s recovery process (Burns-Lynch et al. 2011; Kaliniecka and Shawe-Taylor 2008; Lynch 2011), and reduce re-victimisation (van den Berg et al. 2015).

Given the lack of interventions dealing with the impact of victimisation related to social participation, this study aims to develop an intervention and an accompanying training programme to support professionals to initiate the conversation on victimisation with clients to both address its impact and prevent re-victimisation, in order to reinforce safe social participation, and to improve recovery. In the definition of victimisation, we include both (criminal) victimisation and (anticipated) discrimination.

### Literature Review

A review of the extant literature provided several studies on the prevalence of victimisation and discrimination (Honkonen et al. 2004; Kamperman et al. 2015; Lasalvia et al. 2013; Meijwaard et al. 2015; Teplin et al. 2005; Thornicroft et al. 2009), or on their risk factors, such as homelessness or substance abuse (de Mooij et al. 2015; Goodman et al. 2001; Sells et al. 2003). Studies that described interventions mainly focused on preventing victimisation, for instance, teaching clients to acquire street smarts skills (Holmes et al. 1997; Jonikas and Cook 1993). Studies on the effectiveness of those interventions, however, have not been conducted. Research on anti-stigma interventions is far more extensive and several effective tools exist, such as Narrative Enhancement and Cognitive Therapy (Gronholm et al. 2017; Hansson et al. 2017). Although these interventions provide promising results, they cover only a part of the risks that individuals have to deal with in community life. Moreover, we found no effective interventions on detecting victimisation and its impeding effects on social participation.

As a possible solution to one of the responses to victimisation or discrimination, i.e. demoralisation among service users, in several studies it is argued that focusing on empowerment (positive) rather than reducing self-stigma (negative) is more effective in mental health interventions that support recovery (Bandura 1997; Corrigan 2004). In addition, understanding why a client is demoralised by their previous experiences may remove the barrier to inclusion (Bertram and Stickley 2005; Lynch 2011).

Being recognised as a human being and feeling connected is a fundamental human need; it is helpful to address this in a more structural and methodical way in a mental health care context, as this connectedness or empathy can facilitate productive therapeutic outcomes (Gerace et al. 2018; Reynolds and Scott 1999). In addition, empathy establishes the client’s ‘sense of coherence’, which is the ability for people to understand what happens to them and to find meaning in this, i.e. the way individuals view their life; this has a positive influence on their health and builds resilience (Antonovsky 1987; Eriksson and Lindström 2005). More specifically, being connected is achieved by acknowledging the pain and struggle, and contributes to recovery (Stuart et al. 2017).

Next, we searched for intervention strategies concerning these mechanisms. We elaborated on the concept of ‘dignity of risk’, first articulated by the consumer movement (Perske 1972), which states that every individual has the right to take reasonable risks to progress in life. This is no less true for individuals with SMI. Risk in mental health care was often used in terms of risk management or reduction, in which the professional assessed whether the client posed a risk to their local community (Davison 1997). Indeed, overprotecting and discouraging clients from taking necessary risks may harm their self-esteem and decrease hope and future perspectives. Building upon this positive perspective, Burns-Lynch et al. (2011) developed a guide in which the ‘dignity of risk’ and the client’s personal choices are promoted and elaborated toward a concept methodology of professional work, including shared decision-making, the aim of which is for individuals with SMI to feel community inclusion. They stated: “There is an inherent risk in almost everything we do in our lives. This should not exclude us from participating, but rather ensure that we properly plan to mitigate the harm that can be associated with the various domains and life activities” (Burns-Lynch et al. 2011, p. 17). In this approach, community integration is the road to recovery, *including* promoting the dignity of risk. In each life domain, the client’s goal is formulated through shared decision-making, including the required skills, barriers, and supports. Subsequently, the risks are assessed per domain to determine the appropriate action. This approach, often labelled ‘positive risk management’, has also been promoted by the UK government (Department of Health 2007). It not only promotes a systematic risk assessment, but also propagates a focus on the client’s strengths. To comprehensively implement this positive risk approach, mental health professionals require skills to assess the client’s risks, strengths, and autonomy (Department of Health 2007).

In sum, professionals should probe for the reasons clients hesitate to pursue rehabilitation goals, to identify the possible impact of victimisation experiences. Addressing victimisation experiences is thought to increase the client’s feelings of acknowledgement, improve the working alliance, and create better coping skills for future vulnerable situations [i.e. tertiary prevention (Rüsch and Thornicroft 2014)].

## Methods

### Design and Procedure

**Fig. 1:**
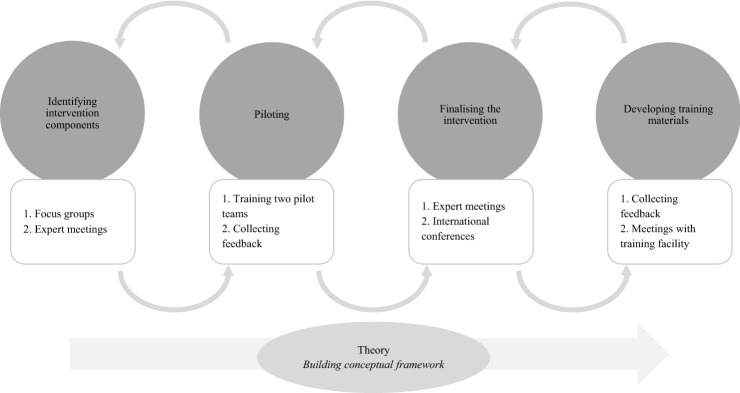
Development process of the intervention

A vital aspect of this development process was its fit with the existing rehabilitation methodologies used by the participating outpatient teams: the Boston University Approach to Psychiatric Rehabilitation (BPR). This person-centred approach was developed to support clients in housing, employment, education, and social contact (Anthony et al. 2002). The victimisation-informed intervention was developed through an iterative process (Fig. 1), using input from the literature review, pilot teams, focus groups, and expert meetings with a core development group and other experts in the field. The core development group consisted of professionals from ‘Rehabilitation’92 (considered to be the leading training facility for the BPR in the Netherlands), researchers, and mental health professionals including experts by experience, and mental health nurses from Flexible Assertive Community Treatment (F-ACT) teams. We did not use formal consensus development methods, such as the Delphi method, but structured discussion was used to reach consensus on the desired content of the intervention (Murphy et al. 1998). The core development group structured information gathered in each development phase and incorporated this into the intervention. No client data were collected during the intervention’s development, so medical ethical approval was not needed. In addition, there were no known conflicts of interest to report.

### Setting

F-ACT teams from two mental healthcare organisations in the Netherlands (Parnassia (formerly Dijk & Duin) and GGzE) and Rehabilitation ‘92 collaborated in the intervention’s development. F-ACT is the leading community mental healthcare programme for people with SMI in the Netherlands (van Veldhuizen and Bähler 2013). Individuals with SMI have a diagnosis according to the DSM-IV, such as schizophrenia or other psychotic disorders, major depression, or personality disorder (American Psychiatric Association 2000). SMI is also defined by illness duration and impact the diagnosis has on one or more major life activities. F-ACT teams are multidisciplinary and comprise mental health professionals such as psychiatrists, psychologists, employment specialists, psychiatric nurses, and experts by experience. One intended benefit of F-ACT is that clients receive care in both periods of stability, where there is a greater focus on rehabilitation, and periods in which they are more at risk of relapse (van Veldhuizen and Bähler 2013). Several team members from each site assisted in developing the intervention, and two teams participated in the pilot. Both pilot teams were certified F-ACT teams according to the official Dutch fidelity guidelines (Bahler et al. 2017).

### Development Process

#### Phase 1: Identifying Intervention Components

Two focus groups were held at the end of 2013 to generate ideas about the content, conditions, and scope of the intervention. Each focus group consisted of around 12 people with varying professional expertise: (specialist) mental health nurses, psychiatrists, psychologists, experts by experience, researchers, and rehabilitation experts. The topics addressed in the two focus groups were: (1) identifying obstacles to social participation related to victimisation and (2) exploring support to address these negative experiences. These topics were addressed from the perspectives of the client, their social network, and a professional caregiver. Cases brought in by the professionals were used to lead the discussion.

Two researchers then translated the results of the two focus groups into the first draft of the intervention. Further elaboration was obtained using a series of four expert meetings with the core development group.

#### Phase 2: Piloting the Intervention

The first version of the victimisation-informed intervention was piloted in two F-ACT teams at the end of 2014. One team from each site was purposively selected; this selection was based on the entire team’s motivation to contribute to developing a new intervention and their affinity with the topic (Greenhalgh et al. 2004), basic knowledge of rehabilitation methods, and having at least one rehabilitation expert and one expert by experience on the team. All team members involved in the pilot teams received training in the intervention. The main goals of the pilot were to identify the barriers to intervention delivery, to examine the feasibility and acceptability of the intervention components, and to monitor the quality and quantity of intervention delivery. We asked the team members to apply the intervention on indication, with the following instruction; explore victimisation in clients within your caseload that have problems in participation. During and after the 6 months pilot period, all team member were interviewed individually via phone (Table 1), and the whole team was consulted to share their experiences of the intervention with the researchers once during a face-to-face meeting. These qualitative data will then be integrated into the intervention.

**Table 1:** Topics of consultations during piloting phase

Were you able to use the intervention (or elements thereof) in your daily job routine? Yes:
With whom, and why that client specifically?
What was the context of the conversation?
How? What was the reason for the conversation? Was there a goal?
Which intervention steps did you take?
What was difficult?
Was it useful? Did it help you?
Are there necessary adjustments to the intervention or conditions?
What was the client’s reaction?
Were you able to use the intervention (or elements thereof) in your daily job routine? No:
No suitable clients; clients did not respond well to intervention
Unsuitable context (of client)
Intervention protocol insufficient or should be adjusted
Insufficient preconditions

#### Phase 3: Finalising the Intervention

Ten expert meetings were organised to translate the findings from the feasibility pilot into concrete adaptations in order to finalise the intervention. The expert meetings varied in their composition but mainly included professionals from the core development group. Additionally, workshops were held at two international conferences: the 2015 European Network of Mental Health Service Evaluation International Conference and the 2015 European Conference on Assertive Outreach (Nijssen et al. 2015a, b). Feedback from workshop participants about the content and form of the intervention was incorporated into it.

The final intervention is described using the template for intervention description and replication by Hoffmann et al. (2014). This template is a 12-item checklist developed as an extension of the CONSORT and SPIRIT statements to provide further guidance for authors regarding the key information to include in trial reports (including name, rationale, materials, procedures, mode of delivery, and infrastructure).

#### Phase 4: Training and Supervision

The core development group generated the training materials, incorporating feedback and insights collected during the previous phases.

## Results

### Results from the Focus Groups, Expert Meetings, and Pilot

This section will present the results per phase of intervention development and will end with a description of the final intervention, entitled the Victoria intervention, which refers to victory and victimisation.

#### Phase 1: Identifying Intervention Components

First, in the focus groups, 21 barriers to social participation were identified and grouped by source (i.e., clients, clients’ social network, mental health professionals). The aforementioned barriers included the lack of belief in the client’s abilities (i.e., by their social network and professionals) and clients’ experiences with unsafe living environments (see Table 2).

**Table 2:** Intervention components based on focus groups

	Perspective		
	Client	Client’s network	Mental health professional
Barriers to social participation	Lack of self-esteem and confidence Fear of relapse Fear of negative reactions from society Negative experiences with participation or negative learning experiences Lack of finances or other facilities Cognitive impairments Positive/negative symptoms Relationship with professional	Lack of belief in client’s resilience Keeping client from rehabilitation Lack of a safe home environment Lack of support from relatives	Lack of belief in client’s resilience Client is not ready for a rehabilitation trajectory Professional wants ‘too much, too soon’ Not connected to the client’s narrative Treatment plan is not specific enough in terms of responsibilities Lack of communication with other organisations Focus on crisis prevention Too few supervision meetings about rehabilitation and recovery Lack of knowledge on rehabilitation methods Lack of (knowledge of) positive risk management
Suggested intervention ingredients	Reduce professional distance		
	Stop filling out the client’s goals and wishes and focus on the client’s narrative		
	Believe in the client’s recovery		
	Increase usage of experts by experience		
	Put the client in the lead		
	Focus on the client’s strengths instead of their weaknesses		
	Incorporate a systemic approach		
	Support system for relatives, focus on perspective of support system		
	Integrate the intervention into existing methods		
	Identify and evaluate risks (‘dignity of risk’)		

Second, participants were asked to develop solutions. One important suggestion was that the intervention should be integrated into existing methods and daily practice. Furthermore, participants suggested that it should aim to identify and evaluate risks in social experiences with a focus on the client’s strengths instead of a focus on signals that precede a relapse. Finally, connecting to the client’s narrative was an underlined intervention component.

In the subsequent expert meetings, participants underlined a focus on both the awareness and dignity of risks, staying connected to the client’s narrative, and targeting the client’s strengths. Furthermore, in terms of practicality, they determined that the intervention should specifically target victimisation experiences, be easy to execute, and include a limited number of ingredients. The experts had some difficulties incorporating the role of relatives into the intervention, aside from focusing on the social network’s role in rehabilitation, and decided to stick to the original aim: develop an easy-to-use intervention that incorporates existing methods by focusing on the interaction with the client.

#### Phase 2: Piloting the Intervention

Overall, the pilot teams were able to use the intervention on a regular basis with several clients in their caseload, but they found it difficult to switch from a problem-focused attitude to development-oriented conversations. They had problems starting the Victoria conversation, especially with clients who initially had no obvious victimisation experiences (e.g., violent assault is often more obvious than discrimination within their family). Furthermore, several mental health professionals were hesitant to use the intervention with clients who suffered from psychotic symptoms and severe substance use because of the client’s distorted sense of reality. Mental health professionals also experienced difficulties with the intervention’s division into two target groups: the group with a high risk of relapse over several life domains and the more stable group. Since clients can switch from one group to the other, it is difficult to determine the starting point of the intervention.

The professionals confirmed that using the intervention led to new insights about their clients, and it helped them better understand why a client had problems with participation. Additionally, it helped the professionals adopt an active listening strategy instead of providing immediate solutions. Interestingly, several experts by experience explained that they had already had these types of conversations with some clients. Finally, the professionals concluded that to successfully implement this intervention, it should be a structural topic in team meetings.

#### Phase 3: Finalising the Intervention

First, indications for the intervention were clarified in the expert meetings, as the pilot team members appeared to have difficulty in recognising the signals that justified beginning the conversation. Second, the intervention targets the entire F-ACT caseload, as problems in social participation (either avoidance or stagnation) are the indication for starting it. Instead of only focusing on clients that have a higher chance of relapse over several life domains, all clients need support in social participation. In this way, Victoria was defined as a preamble or restart intervention for rehabilitation methods. Third, the content of the intervention was converted into delineated steps with a more clearly defined start and finish. This also adds to the better determination of the intervention’s starting indication. There was a need for clear options after finishing the initial conversation, as not all problems relating to social participation are due to victimisation experiences. This was incorporated into the last step. Finally, participating in international conferences supported the notions that mental health professionals often underestimate the prevalence of victimisation, and that there should be a focus on the role of the social network in tackling victimisation as a barrier for participation. This is incorporated in the ‘clarifying context’ step.

#### Phase 4: Training and Supervision

Basic training in a rehabilitation methodology is fundamental, since experiencing difficulties in rehabilitation trajectories is a reason to start the Victoria intervention, with the intention of exploring whether victimisation is blocking participation and getting back on track to (re)start rehabilitation.

Intervention training includes three half-day sessions provided by two trainers, one of whom is an expert by experience (as suggested in the expert meetings). These sessions focus on explaining the background of the intervention, including some theory, and explaining the four steps. To ensure the comprehensive implementation of the Victoria intervention, pilot teams suggested incorporating it into team meetings. As such, the first training session and supervision meeting includes a team brainstorm about ways to secure the intervention in the daily job routine on individual and team levels. The second and third training sessions include practicing in small groups by using role-play in which real-life cases are used and discussed; several fictitious cases are available. To ensure that professionals use the intervention in practice, supervision meetings with a Victoria trainer every 6–8 weeks form part of the training.

To assure fidelity, it is important that training sessions be similar across teams. Therefore, concrete materials were developed for train-the-trainer education, including a short educational film showing a good example of a Victoria conversation with a client. The film includes an expert by experience in conversation with a Victoria trainer. Other training materials are the manual and a shorter handout in two sizes: one to take along and a poster to display in the office.

### Description of the Victoria Intervention

#### Case Vignette

To better understand the procedure of the Victoria intervention, a sample case is given below. This is based on cases brought in by mental health professionals during the development phases.

Tom is a 33-year-old man with a long history of mental healthcare and drug abuse. He lives with his mother in a small apartment. She cooks and takes care of the household. Tom has difficulty getting up in the morning and has no structured daytime activities. Lately, he hardly gets out of the house at all. His opinion is that by staying in the house, he keeps out of trouble. In one of the appointments with the case manager, the case manager explores why Tom keeps having issues with getting out. After a while, Tom admits that he was harassed by one of his former friends, who wanted money, which Tom did not have. Tom managed to get away, but after this incident, he lacked the confidence to go out more.

#### Step 1: Exploring Social Participation

**Fig. 2:**
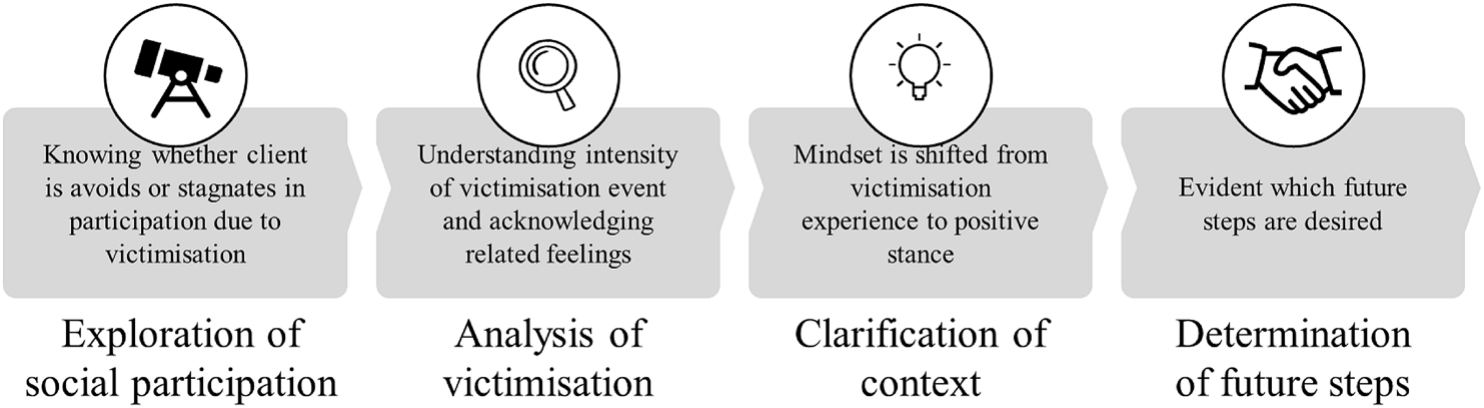
The four steps and the goals of the Victoria intervention

The *Exploring* step of the Victoria intervention (presented in Fig. 2) incorporates the elements and skills taught in the ‘goal attainment module’ of BPR and involves the evaluation of activity and satisfaction in the following life domains: housing, social contacts, education, and work. These domains are part of the rehabilitation methods used (the BPR in the pilot teams) (van Veldhuizen and Bähler 2013). With the client, the mental health professional determines whether the client is avoiding activities or whether the desired progress on these domains is stagnating. Specific to Victoria is the exploration of the possible role of recent victimisation experiences in this. When problems regarding social participation are not linked to victimisation (e.g. not having the right education for a desired job), the professional may (re)start a rehabilitation action plan with the client.

#### Step 2: Analysing Victimisation Experiences

While recent victimisation appeared to play a hindering role in participation during step 1, the second step is *Analysing victimisation experiences* by addressing who, what, where, and when. The professional uses a client-centred approach (Rogers et al. 2006) to effectively understand the intensity of and feelings related to this specific experience. In line with conversation techniques standard across mental health practice, or in BPR training, it is crucial that the professional uses an active listening strategy to support the client in elaborating on his/her victimisation experience. Adding to these conversation techniques is a narrative approach in which the professional acknowledges the pain and struggle of the client’s victimisation experience (Rogers et al. 2006). The overall goal in this step is to get a full picture of the event, to recognise and acknowledge feelings related to it, and to understand why it made the client stagnate in participating in, for example, paid or voluntary work, and daily or leisure activities.

#### Step 3: Clarifying the Context of Victimisation Experiences

The third step entails *Clarifying the context* of the victimisation experiences and incorporates elements from the concept of the ‘dignity of risk’ (Perske 1972) and the positive risk approach (Burns-Lynch et al. 2011). The professional works with the client to examine the motive for engaging in the situation in which the victimisation occurred. If the underlying desire or wish is clear, the client explains to the professional how they would have wanted the situation to go and what they had hoped to achieve by engaging in this situation. Again, it is critical that the professional use an active listening strategy, as the client’s story and perceptions are crucial to fully understanding their reasoning and wishes (following the client-centred and narrative approach). This step intends to shift the mind-set from the victimisation experience back to a more positive stance and change it into a learning experience. Application of the Victoria intervention is personalised, as one client may need and want several conversations and another may be satisfied with one or two, so several future steps are plausible.

#### Step 4: Determining Future Steps

The fourth and final step of the intervention is *Determining future steps* based on the results of steps 2 and 3*.* If both the client and the professional agree that the discussed experience is indeed an important obstacle to social participation (i.e., they become more aware of the barriers and the client feels acknowledged), then the next step is to (re)start the BPR rehabilitation action plan to work on the client’s original rehabilitation goals and wishes from before the victimisation experience, working to conform the principle of dignity of risk, and the positive risk approach, incorporating a risk management plan in order to prevent re-victimisation. If the victimisation experience was very intense, the professional should use the ‘Trauma Screening Questionnaire’ (TSQ) to investigate whether trauma-focused treatment is needed (de Bont et al. 2015). The ten items of the TSQ are answered ‘yes’ or ‘no’; if six or more items are answered ‘yes’, trauma treatment may be beneficial and is advised in the Victoria intervention. When the victimisation is still going on, mental health professionals would first discuss signs of victimisation with the client, and in consultation with the client, the professional can consult family or friends. Following Dutch law and regulations, mental health professionals have the legal duty to report domestic violence and child abuse, violence and other crimes within mental health care organizations, and victimisation in other settings when there is serious damage or danger for the client or others to expect (Ministry of Health Welfare and Sport 2014; WGBO 2019; Wkkgz 2020).

#### Mode of Delivery and Infrastructure

The intervention is intended for individuals with SMI that experience problems with social participation; it was developed in such a way that it can be used by every professional in community mental health teams. In practice, it is largely used by professionals with their own caseloads (e.g., psychiatric nurses), but experts by experience can also use it due to their narrative working practice.

The Victoria intervention was developed to be used during regular meetings with clients, at the client’s home or at the outpatient team’s location. It is able to be integrated into regular work processes, and familiar conversational techniques are used to carry out the steps. Preferably, those steps are integrated into regular sessions where other issues are also discussed, rather than in separate appointments to solely discuss victimisation experiences. It should be noted that the first step, exploring social participation, should be repeated regularly as part of standard rehabilitation. As clients’ situations change over time, so do difficulties with social participation. Furthermore, it is advised that the professional has an agenda for these appointments and not be swayed by issues of the day.

## Discussion

This paper outlines the development of a victimisation-informed intervention that aims to expand the awareness and acknowledgement of victimisation experiences and provide concrete professional tools in working with such experiences. The intervention aims, in this way, to encourage future safe social participation of people with SMI.

As a result of the feedback from the pilot group the victimisation-informed intervention is positioned as a preamble intervention used as an add-on to existing rehabilitation methods. It builds on the phase in which the personal goals are defined in that it identifies victimisation as a reason behind why people may stagnate in their goal attainment and thus participation. It is used as a precursor to identify if other trajectories or interventions on victimisation are required, including trauma-focused treatment or additional Victoria conversations. One could also think of follow-up interventions which enhance participation that are focused on social support (Castelein et al. 2008), anti-stigma interventions (Thornicroft et al. 2016; Yanos et al. 2011), or supported employment (Becker and Drake 1994). If the family has an impeding effect on the autonomy and participation of the client, family interventions may be required (Pharoah et al. 2010). Moreover, with the recent developments in interventions such as Resource Group Assertive Community Treatment or resource groups (Tjaden et al. 2019), further development should focus on integrating the intervention in those methods, as we know that the social network has a great influence on both victimisation and rehabilitation.

Our Victoria intervention was inspired by the concept of ‘dignity of risk’ (Perske 1972) and positive risk management (Burns-Lynch et al. 2011), which have both recently attracted attention. A recent scoping review on recovery shows that it is important to maintain a balance between taking risks and safety in recovery processes, while still empowering clients (van Weeghel et al. 2019). Difficulties are inherent within a recovery process and should therefore be incorporated in recovery-oriented mental health services (Stuart et al. 2017). As Sweeney et al. (2018) argued, this involves a shift from professionals thinking ‘what is wrong with you?’ to ‘what has happened to you?’, or move away from ‘managing risk’ to ‘promoting safety and opportunity’ (Perkins and Repper 2016). A recent article of Jones (2020) adds to this notion by suggesting a more positive stance towards risk. Moreover, risk and recovery go hand-in-hand. Slade et al. (2013, p. 52) agree and argue that: “the largest contribution by mental health services to supporting recovery may come from enabling the empowerment of patients to experience the full entitlements of citizenship”.

Adopting victimisation-informed care involves a shift in professionals’ attitudes from being more symptom focused toward a more narrative-type approach that increases the awareness of and attention to victimisation and may present several implementation challenges. First, professionals in mental health community settings often have to deal with large caseloads (in ACT and F-ACT teams: ten staff members for 100 to 200 clients) in which psychiatric crises, violence, nuisance, or urgent housing issues often draw attention away from rehabilitation needs. To overcome this potential barrier, we developed the Victoria supervision meetings as part of the training to support these professionals in overcoming this potential pitfall. Furthermore, the intervention’s small and simple nature and its use as a preface to rehabilitation methods should contribute to its easy and frequent usage in daily practice. Professionals do not need to acquire an entire new skill set, as the conversation techniques in the intervention are standard practice in their education (van Veldhuizen et al. 2008). Second, the Victoria intervention requires a new perspective: a delicate balance between client safety and letting them take risks as part of their recovery process; embracing this paradigm will take time. Peer workers may have a pivotal role in this, as they understand the perspectives of both the professional and the client (Davidson et al. 2012).

### Strengths and Limitations

To the best of our knowledge, this study is the first to describe an intervention that addresses victimisation and discrimination experiences, which are hardly addressed but highly prevalent in clients with SMI on an almost daily basis, and form large barriers in their recovery. In contrast to current practice, this intervention aims to (1) address the experience in order to enhance acknowledgement, as well as (2) stimulate healthy and safe social participation. In this way, the intervention aims at enhancing current rehabilitation practices and recovery-focused working. Another strength is having used the extensive development period of 2 years, which allows for the intervention to be based on a range of findings, including information from several expert meetings, pilot testing, and focus groups. Its other strengths include the involvement of a range of stakeholders throughout the development process (professionals, rehabilitation experts, researchers, managers, clients, etc.) and the usage of conversation techniques that are standard practice in education which allow for an easier integration into daily practice.

Our study also has certain limitations that should be acknowledged. First, even though 2 years is enough for the development of an intervention, it is less generous for the testing of a solid implementation strategy. This study led to a training protocol, including three training sessions and regular supervision meetings with skilled trainers, and intervention tools to be used in daily practice. The next step would be to get experience in implementation, including further (graphical) development of the materials, creating awareness of the need for the intervention, and a sense of shared responsibility in mental health professionals and management to recognise and address victimisation. Second, during the piloting stage we did not include any additional quantitative data collection, which could have been informative about how many and which type of clients received the intervention. Finally, a fidelity instrument would be relevant to stimulate accurate use of the intervention.

## General Conclusion

Developing the Victoria intervention is a first step in addressing the victimisation experiences that hamper many people with serious mental health problems in their social participation and general wellbeing. This intervention incorporates the recognition and acknowledgement of the victimisation experiences that individuals with SMI face in their recovery process and provides both professionals with concrete tools to work on victimisation and clients with new perspectives on rehabilitation. Next steps will be the evaluation of the effects of this intervention on social participation and victimisation in a first (pragmatic) cluster randomized controlled trial, and the implementation process will be examined in a process evaluation.
